# Langmuir Monolayer Techniques for the Investigation of Model Bacterial Membranes and Antibiotic Biodegradation Mechanisms

**DOI:** 10.3390/membranes11090707

**Published:** 2021-09-14

**Authors:** Monika Rojewska, Wojciech Smułek, Ewa Kaczorek, Krystyna Prochaska

**Affiliations:** Institute of Chemical Technology and Engineering, Poznan University of Technology, Berdychowo 4, 60-965 Poznań, Poland; wojciech.smulek@put.poznan.pl (W.S.); ewa.kaczorek@put.poznan.pl (E.K.); krystyna.prochaska@put.poznan.pl (K.P.)

**Keywords:** antibiotics, Langmuir monolayer, bacterial membrane, LPS, phospholipids, antibiotic resistance

## Abstract

The amounts of antibiotics of anthropogenic origin released and accumulated in the environment are known to have a negative impact on local communities of microorganisms, which leads to disturbances in the course of the biodegradation process and to growing antimicrobial resistance. This mini-review covers up-to-date information regarding problems related to the omnipresence of antibiotics and their consequences for the world of bacteria. In order to understand the interaction of antibiotics with bacterial membranes, it is necessary to explain their interaction mechanism at the molecular level. Such molecular-level interactions can be probed with Langmuir monolayers representing the cell membrane. This mini-review describes monolayer experiments undertaken to investigate the impact of selected antibiotics on components of biomembranes, with particular emphasis on the role and content of individual phospholipids and lipopolysaccharides (LPS). It is shown that the Langmuir technique may provide information about the interactions between antibiotics and lipids at the mixed film surface (π–A isotherm) and about the penetration of the active substances into the phospholipid monolayer model membranes (relaxation of the monolayer). Effects induced by antibiotics on the bacterial membrane may be correlated with their bactericidal activity, which may be vital for the selection of appropriate bacterial consortia that would ensure a high degradation efficiency of pharmaceuticals in the environment.

## 1. Antibiotics in the Natural Environments

**Figure 1:**
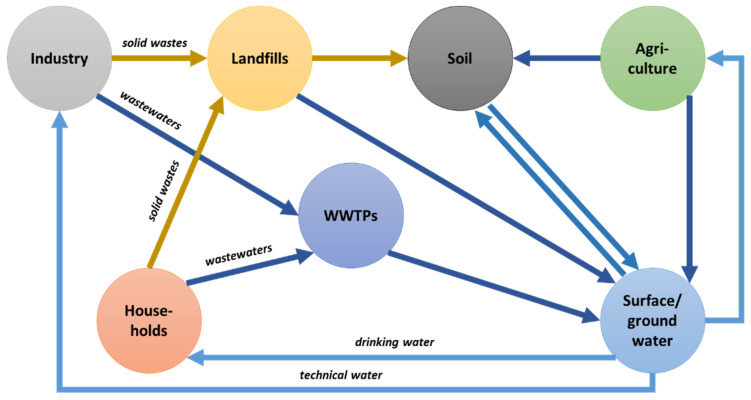
The migration pathways of antibiotics to the environment (according to [4]).

After the discovery of antibiotics by Alexander Fleming in 1929, the expanding pharmaceutical industry commercialised over 160 new antibiotics and semi-synthetic derivatives in the years 1940–1970 [1]. As a result of their high efficacy, antibiotics have become the first-choice treatment of many infectious diseases, and their use has led to a significant reduction of morbidity and mortality related to common microbial infections. Antibiotics are applied on a large scale, their worldwide consumption is estimated in the range between 1 × 10^5^ and 2 × 10^5^ tons annually [2,3]. Unfortunately, the excessive spreading of antibiotics in the environment and their strong impact on microorganisms, together with long-term negligence of appropriate regulation of drug pollutions on local and global scales, have led to serious disruptions in the proper functioning of many ecosystems. Moreover, the growing medicinal needs and, consequently, increasing consumption of pharmaceuticals have also notably contributed to this problem through the release of high amounts of pharmaceutical and hospital waste into the aquatic ecosystems (Figure 1).

Many pharmaceuticals are only partially metabolised by human and animal organisms, e.g., approximately 70% of antibiotics pass the human digestive system unaltered—they are excreted via urine [5] and eventually end up in sewage [6,7,8].

In particular, heavy contamination of water is caused by orally administered medications, as much as 90% of which is excreted from the human body and consequently enters the aquatic systems [9]. Similarly, veterinary medicines generate water contaminations because the administered antibiotics are not completely absorbed by animal organisms [10,11,12]. As a result, the large-scale production of livestock is another major source of antibiotic contamination of the environment. In particular, an alarming increase in the use of antibiotics is observed in aquaculture, which is now perceived as the fastest-growing food sector in the world [13,14,15]. The exact amount of antibiotics used in this food sector is difficult to assess, but it certainly is much higher than the amount used by hospitals [16,17,18]. Many authors have reported that antibiotics applied in animal husbandry account for approx. 60% of the total antibiotic production, and this trend is growing steadily [19,20]. Among the broad spectrum of antibiotics, tetracyclines and penicillins are the most commonly used, followed by sulphonamides [21]. However, high concentrations of other compounds, including beta-lactam antibiotics, aminoglycosides, phenicols, macrolides, and glycopeptides, are also observed in the environment [22].

Unfortunately, the currently operating wastewater treatment plants (WWTPs) have not been designed to completely remove antibiotics. Consequently, antibiotic molecules are increasingly found in terrestrial, freshwater, and marine environments [11,23], accumulating in the environment and leading to poisonings of many useful microorganisms [24]. The excessive presence of antibiotics in the environment also leads to an increased frequency of finding antibiotic-resistant bacteria (ARB). The phenomenon of antibiotic resistance results in serious disturbances in the environmental balance and is now referred to as ‘antibiotic resistance pollution’ [25]. First of all, antibiotic residues promote the occurrence of antibiotic resistance genes (ARGs) and contribute to the development of new defence mechanisms by microorganisms. In consequence, this effect also has an important impact on public health [5,8].

Moreover, the latest observations have shown a high number of ARGs in the environment, which is explained by the fact that many species of bacteria have adapted to the use of antibiotics as a source of nutrients, which naturally leads to enhancement of the bacterial resistance to these substances. Currently, it is believed that the development of antibiotic resistance is caused by mutations and horizontal gene transfer, which have been driven by the selective pressure of antibiotics applied in therapeutic processes. As has been proven, this selective pressure associated with antibiotic pollution affects the overall microbial community and results in changes in its composition, which tend to favour an increase in Gram-negative (G-) bacteria, as opposed to Gram-positive (G+) bacteria. These disturbances in the natural ratio of microorganisms in the ecosystems may result in a loss of biomass and reduction in microbial activity in terms of nitrification, denitrification, and respiration in soil [26,27,28], or may lead to disruption of carbon cycling and primary productivity in aquatic environments [29]. Finally, antibiotics disturbing the balance of microbial communities can also lead to an increased abundance of parasites and pathogens in both soil and water environments [26,30].

It should be emphasised that the presence of antibiotics in the environment is associated with the spread of antibiotic resistance among microorganisms, primarily in non-pathogenic ones, and through them also to pathogenic species. However, at the same time, there is a process of their biodegradation (albeit slow) by adapted microorganisms which possess relevant metabolic pathways [31]. It is worth noting, however, that for the most part, drug resistance mechanisms are not the same as drug degradation mechanisms. Antibiotic resistance is usually associated with blocking access of the antibiotic to the cell through the production of protective extracellular compounds. In contrast, biodegradation of an antibiotic, similar to any xenobiotic, depends on the genetic material of given bacterial-strain-encoding enzymes capable of metabolising the molecule, which sometimes (but not always) may be the cell’s strategy for survival in the presence of the antibiotic [32]. In many cases, it is observed that biodegradability does not preclude sensitivity to a given antibiotic, as confirmed by toxicity tests of a given compound against different strains at different concentrations of the contaminant [33].

The degradation products of antibiotics by hydrolysis or photolysis processes are also dangerous because they become the transformation products (TPs) which may also change the structure of bacterial communities. Many antibiotics are directly photolysed in the environment, for instance, penicillins and tetracyclines [30]. Jiao et al. [34] have shown that the products of phototransformation of antibiotics may be more toxic to microorganisms than the original drugs. There are results that have already shown the toxicity of metabolites of some antibiotic classes, e.g.**,** sulphonamides, tetracyclines, and fluoroquinolones [35]. Moreover, some metabolites, such as N4-acetylsulphapyridine and N4-acetylsulphadiazine, have been demonstrated to be more toxic than their parental compounds [36]. Sulphonamides (SAs), macrolides (MLs), tetracyclines (TCs), and quinolones (QNs) are the dominant antibiotics in surface waters which are mainly related to aquaculture and the emission of domestic sewage [37]. In the soil, the antibiotics usually occur in lower concentrations than in water [38]. Triclosan, sulphadiazine, and trimethoprim are most often found in soils.

In light of the above data, the need to analyse the biological degradation of antibiotics, both as a natural spontaneous process and as a strategy to effectively remove these compounds from wastewater or contaminated soils, has become more urgent.

## 2. Methods to Remove Antibiotics from the Environment

Conventional WWTPs are not sufficient to eliminate the microcontamination with antibiotics [5,39,40,41]. Therefore, the development of innovative and advanced technologies for post-WWTP clean-up is urgently needed [7,42]. The most popular solutions include biological treatments, ozonation, Fenton and photo-Fenton processes, semiconductor photocatalysis [43], chlorination, electrochemical oxidation, adsorption [44,45], and membrane filtration [46]. These methods can achieve various removal efficiencies of antibiotics present in surface and ground waters as well as wastewater, but most of them have numerous limitations. The most commonly used adsorbents in wastewater treatment are activated carbon, zeolites, clays, and agricultural wastes [47,48]. However, adsorption only transfers the antibiotics from the liquid to the solid phase, without their complete degradation [49]. On the other hand, membrane processes demand a high-quality influent, which is often accompanied by secondary pollution caused by the residual concentrated water. Although the technical feasibility of membranes has been proven, high investment and operational costs limit their widespread application [5]. It should be emphasised that numerous pharmaceuticals are not separated on the membrane during an ultrafiltration process. On the other hand, the main disadvantages of electrochemical oxidation include its high operating cost, production of oxyhydroxides precipitates, and the necessity to recover the dissolved catalyst [5].

The biological methods are considered as most convenient bioremediation solutions because they require less energy and chemical inputs [50]. These processes use the ability of microorganisms to uptake organic matter from wastewater. The organic compounds degraded by bacteria are consumed as energy and carbon/nitrogen sources [51]. However, these processes may fail in the case of some specific contaminants, such as pharmaceuticals. Anaerobic biological treatment cannot effectively remove aqueous antibiotics because of their bacterial resistance, resulting in their incomplete degradation or adsorption to sludge.

In view of the above, several improved biological processes, sometimes coupled with physicochemical techniques, have been proposed to remove antibiotics in WWTPs [52]. Many research reports and reviews have described the use of conventional-activated sludge (CAS) and membrane bioreactors (MBR) to remove antibiotics.

In the case of removal of antibiotics by biological processes in WWTPs, there are still many unsolved questions regarding the biodegradation pathways and biological factors—microorganisms and enzymes—involved in each step of the treatment. Moreover, the complexity of the environment in which the biodegradation process is carried out simply does not allow to precisely predict the treatment efficiency. The presence of other contaminants may antagonistically or synergistically influence the removal of a particular antibiotic [51].

In order to better plan and understand the biodegradation processes, the interaction of microorganisms with antibiotics must be known. The questions that need to be answered include the reasons why one group of microorganisms is capable of effectively degrading antibiotics and another is not, or what is the effect of the biological membrane composition on the degradation of substances by microorganisms.

Therefore, it is also important to investigate the influence of the environment on the interaction between the bacterial membrane surface and antibiotics. The carbon and energy source, i.e., the biodegradable compound, penetrates through the cell membrane. Its penetration is one of the stages limiting the bioavailability of the compound and, consequently, its biodegradability. Therefore, understanding the interaction mechanisms between the antibiotic and the phospholipid membrane has serious consequences for a better design of the biodegradation processes of emerging contaminants, such as antibiotics.

Moreover, another aspect of this type of research should be noted. It is precisely the transport of an antibiotic inside the cell that is one of the key parameters determining its effectiveness as a pharmaceutical. Irrespective of the type of antibiotics, i.e., those directly affecting the cell wall and the cell membrane (such as penicillin) or those that act inside the cell (such as streptogramins), the interaction with the cell membrane may be a step limiting or maximising the effectiveness of the biocidal action [53].

It should be emphasised, however, that the influence of a contaminant (e.g., an antibiotic) on the biological membrane may be of physicochemical nature, related to the phenomena occurring on the phase surface, or of a biochemical nature, i.e., changing the active transport through the membrane by transport proteins. To verify which of the processes is decisive, it seems to be extremely useful to conduct measurements on a model phospholipid membrane, which is a biomimetic system imitating the actual membrane to a certain extent. By comparing the results obtained for the model system and the real system, it becomes possible to confirm or exclude the role of interphase processes and the direct interaction of antibiotics with the phospholipid layer [54,55]. Nevertheless, model membrane testing is also challenging. Firstly, because of the necessity to select a proper membrane composition and, secondly, because of the adjustment of appropriate measurement techniques.

It should be emphasised that without a proper understanding of the mechanisms of antibiotic penetration inside the cell, it will be extremely difficult to predict and control this process. Considering the role of active transport through the cell membrane, the issues of physicochemical interactions with the cell membrane should not be neglected. In this context, the use of appropriate research methods, such as the Langmuir monolayer technique, is extremely important.

## 3. Modelling of Cellular Membrane

Living systems possess biological membranes which consist of many oriented molecules [56]. These structures are fundamental components of the cell because they separate the internal contents of the cell from the surrounding environment and play a vital role in transporting the material between various organelles and the plasma membrane. The oriented lipid molecules consist of hydrophilic head groups and hydrophobic tails. Charged atoms in the hydrophilic head enable the formation of electrostatic bonds with the polar water molecules, while the non-polar groups in the hydrocarbon tails are averse to exposure to water. For this reason, the lipid molecules contacting with water arrange themselves in the opposite orientations, forming bilayers that effectively shield the tail groups from the surrounding aqueous solution.

In general, membranes form closed structures in which the lipid molecules are free to diffuse on the surface. As a consequence, the membranes are characterised by a long-range orientational order but lack a positional order. In effect, membranes behaviour is fluid-like, and thus, they may be regarded as two-dimensional liquid crystals. Bilayers are considered a flat configuration which seems the most preferred due to their homogeneous composition and identical environment on either side [57]. However, in the presence of different species of lipids or interaction with other biological structures, a locally curved state becomes the preferential configuration. This effect is observed in the presence of proteins which are integral parts of biological membranes, and their presence in the membrane influences the shape of the membrane [58,59] and determines the transport of material into the cell, which is especially important in the case of endocytosis [57,60]. Biological membranes are composed of numerous proteins at concentrations reaching as high as 25% area occupancy, resulting in crowded environments [55]. Over the last years, it has become increasingly apparent that lipids within biological membranes have intricate relationships with membrane proteins, and their interaction with the membranes is fundamental to comprehend the structure of biomembranes and many physiological phenomena [61,62].

Bacterial membranes include various lipids, mainly phospholipids listed in Table 1. Among them, there are three major lipids: phosphatidylethanolamines (PEs), phosphatidylglycerols (PGs), and cardiolipin (CL) [63]. The phospholipids differ in the structure of polar heads; the glycerol polar head in PG is larger than the ethanolamine group in the PE molecule [63]. Cardiolipin (diphosphatidylglycerol) is an anionic phospholipid similar to PG, but it consists of two phosphatidyl moieties linked by glycerol and contains four chains within the molecule. Bacterial membranes are very diverse in terms of the content and ratio of individual phospholipids. Moreover, bacteria themselves exhibit the ability to change the composition of the membranes in order to best adapt to environmental conditions [34]. This adaptation process also promotes an increasing variety of membrane compositions and formations of membranes with different physicochemical properties. According to literature reports [41,63], the membranes of Gram-positive species are enriched in anionic lipids, or they are completely devoid of PE. On the other hand, phosphatidylethanolamine, PE, is dominant in the inner membrane of Gram-negative bacteria [40], while anionic lipids such as PG or CL are minor components. Moreover, in Gram-negative bacteria, the envelope surrounding the cells consists of an outer membrane (OM) mainly containing LPS in the outer leaflet and only various species of phospholipids in the inner leaflet (an inner membrane, IM). Thus, in Gram-negative bacteria, drugs have to cross the additional LPS barrier. In this case, antibiotics transport is enhanced by porin channels, which allow the antibiotics to be transferred to the periplasmic space, where the therapeutic substances can bind to their targets and develop a biological activity. Porins are barrel proteins that pass through the entire thickness of the membrane, and they are responsible for the passive diffusion of molecules, also including the influx of antibiotics (mostly hydrophilic and smaller than 1500 Da) [64].

Biological membranes are complex systems; therefore, in the last century, many model membranes have been developed to study the properties, structure, and processes that occur within them [66]. Moreover, many models have been designed to describe the transport mechanism of various natural or synthetic compounds (such as surfactants, peptides, proteins, and drugs) penetrating the membrane. The most common biomimetic systems used to characterise protein–lipid and drug–lipid interactions are lipid vesicles (liposomes), lipid bilayers (SLB), and lipid monolayers [67,68,69]. All these systems mimic the lipid arrangement of natural cell membranes. However, each of them exhibits some advantages and disadvantages (Table 2).

The most widely used artificial membranes are vesicles which consist of a lipid bilayer and entrapped aqueous solution. Liposomes can vary in size and lamellarity depending on their preparation. Multilayer vesicles (MLVs), which can incorporate any type of lipid, are very easy to produce. However, the main problem during the analysis of such systems is their multilamellar nature. The presence of many internal compartments limits the use of multilamellar liposomes, especially when transport mechanisms and permeability are to be studied. This structure makes them improper for the study of lipid asymmetry or transport across the membrane because it is difficult to define an exterior and an interior of the vesicle. Moreover, the large size of these objects makes them unsuitable for structural study by NMR [70]. For this reason, unilamellar vesicles are more popular, despite the fact that they are generally less stable than MLVs. The major advantages are associated with the easily defined inner and outer layers of the liposome, and the transport of a substance across the membrane can also be analysed. Depending on the diameter, the following can be distinguished: giant unilamellar vesicles (GUVs, range from 100 nm to several hundred µm), large unilamellar vesicles (LUVs, 20–100 nm), and small unilamellar vesicles (SUVs, down to approximately 25 nm) obtained by ultrasonication [71].

Moreover, bilayered micelles (bicelles) and nanodiscs are used to study biomembranes properties, primarily for the study of membrane peptides and proteins. According to the literature [72], bicelles are formed by mixing long- and short-chain lipids to obtain a membranous disc. They are most commonly composed of dimyristoylphosphatidylcholine (DMPC) and dihexanoylphosphatidylcholines (DHPC) [73]. Furthermore, cholesterol, charged lipids, or surfactants can be incorporated into their structure [73]. This is beneficial because it allows bicelles to mimic many complex biological systems such as prokaryotes, mitochondria, erythrocytes, myelin, neurons, and skin. Bicelles have different morphologies, but the most popular is the disc topology with a diameter of a few tenths of nanometres. Long-chain phospholipids form the planar region of a disc which constitutes a favourable environment to study molecular interactions between the structure of membrane peptides and proteins. They have magnetic properties which are very useful to determine their structure by NMR [72] and allow researchers to widely apply bicelles for understanding dynamical changes of membranes properties during their interactions with many substances, especially with peptides or proteins [72]. On the other hand, the temperature and the composition of bicelle influence the thermodynamic stability of its structure and thus limit its applicability [74]. Moreover, bicelles have relatively high fragility, especially during the embedding of substances into bicelles when the disk structure may become destabilised as a result of competition between incorporated peptides and the small chain lipids. In consequence, membrane systems with a more stable structure than bicelles and nanolipoproteins (NLPs) have been sought. These structures are also known as nanodiscs. Nanodiscs are nanometric lipid bilayers which consist of a non-covalent assembly of a phospholipid surrounded by a ring of a dimer of lipoprotein such as apolipoprotein. The main advantage of this biomimetic system is that membrane proteins associated with nanodiscs form highly stable structures that enable the study of lipid–protein interactions at higher temperatures and over a longer period of time using the NMR technique. The size and composition of lipids can be easily regulated in nanodiscs; therefore, these structures constitute a very convenient environment for studying the interactions of lipids and membrane proteins with biological substances [72,75].

Reconstructions of lipid vesicles based on typical procedures allow obtaining equal lipid composition in the two leaflets and randomly oriented proteins, in contrast to very asymmetric native membranes, in which the asymmetry of lipid membrane is maintained by embedded metabolic enzymes and proteins. Therefore, the main limitation of artificial lipid vesicles has been the general lack of lipid asymmetry. Currently, asymmetric lipid vesicles are formed by lipid exchange, i.e., mixing of vesicles composed of the desired inner leaflet lipids with donor vesicles which contain an excess of lipids that build the outer leaflet [75,76]. Other methods include, e.g.**,** vesicles involving the use of lipids dispersed in oils, use of pH gradients, which achieve some asymmetry due to translocation of anionic lipids to the side of the membrane exposed to high pH, or outer leaflet headgroup exchange via phospholipase [76].

The liposomes have been used to generate supported lipid bilayers (SLBs). Supported lipid bilayers can be formed by the fusion of liposomes during adhesion to the surface. This type of bilayer has the disadvantage that the interaction between one lipid monolayer and the support is very strong and, as a result, possibly impacts the physicochemical properties. On the other hand, the planar solid support can stabilise the very fragile structural unit of the fluid bilayer membrane which allows the use of a wide range of surface-analytical tools for analysis. Other attractive methods to obtain SLBs are the Langmuir–Blodgett (LB) film transfer and Schaefer dipping [77,78]. Both methods ensure the formation of a bilayer with precise control over the composition and packing density of the lipid film. However, some problems can occur during the incorporation of less stable proteins because the system is formed at the air–water interface and requires exposure to air for a period of time. Therefore, some proteins, especially toxins or antibiotics, are incorporated after bilayer formation. Moreover, hydrophilic substrates (such as silicon) may interact very strongly with the first layer and, as a result, modify the fluidity of the biomimetic membrane. A biological membrane is a dynamic, fluid system, wherein molecules have translational freedom; therefore, the presence of interactions with the substrate may limit the mobility of phospholipid molecules within the plane of the membrane and significantly impact the interactions of the membrane system with other biological substances [78]. This tethering effect depends on the nature of associated biomolecules with the bilayers system and sometimes may become a major problem to mimic the behaviour of natural membrane. The free-supported phospholipid LB films are prepared to solve this problem. These systems consist of double bilayers—the three first layers are deposited by the LB method and the last one by LS which leads to the immersion of the substrate in the water subphase at the end of the transfer. As a result, it is possible to obtain a system in which two bilayers are separated by a thick hydration layer. The second bilayer (‘free bilayer’) corresponds to a highly hydrated membrane floating above the first one and is a suitable model that simulates the biomembrane [78]. The production of asymmetric phospholipid bilayers, also called alternate-layer LB films, has become an interesting approach to develop biomimetic systems. The simplest way to obtain such a system is to raise the substrate through a monolayer of one material (consisting of molecules A) and then lower the substrate through a monolayer of a second substance (containing molecules B). Asymmetric membranes have also shown specific properties (such as the pyroelectric effect) due to the lack of a plane of symmetry in their structure. Currently, they are considered to be very promising and innovative systems that will enable further understanding of the interactions of biological compounds with the membrane. It should also be emphasised that supported bilayer membranes are applied in many biosensors based on biomimetic membranes [79]. The Langmuir technique is an easy method to form biomimetic systems in case of which it is possible to control lipid composition, molecular packing, physical states, lateral pressure of membrane, and experimental conditions as temperature or pH. The phospholipid monolayers formed by the Langmuir technique are two-dimensional asymmetric structures, and they possess planar geometry. A big advantage of the Langmuir monolayer over the vesicle or liposomes is associated with the well-defined, stable structures and the possibility to precisely control the membrane composition and the molecular areas of lipids [80]. However, the Langmuir monolayer can be used as a model of membrane leaflet, so this biomimetic system is useful to study processes that occur at the membrane surface. Therefore, this model is primarily used in the study of intermolecular behaviour, the effect of a particular component on the membranes, the interactions of biomolecules on the membrane, and also the impact of some parameters such as pH, ion strength, or temperature on the biomembrane. Moreover, Langmuir lipid monolayers are also used to mimic many biological features observed in the case of natural cells, such as lipid rafts and the interaction of proteins with biological membranes.

The main limitations of the presented models are associated with the fact that they do not capture the whole complexity of biological membranes. The simplification of the membrane system is essential for the investigation of specific interactions at the molecular level in the membrane structure. On the other hand, it can also interfere with the accurate understanding of some membrane functions.

## 4. Application of the Langmuir Monolayer Technique to Form Biomimetic System

**Figure 2:**
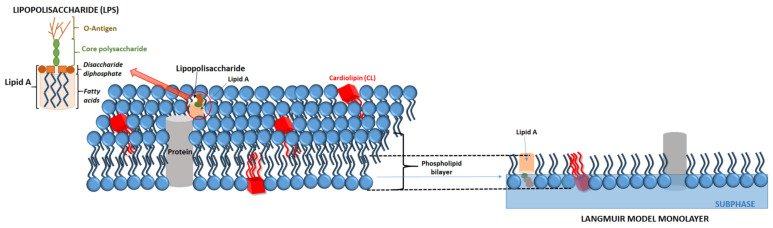
A Langmuir monolayer as a model of bacterial membrane leaflet.

The Langmuir technique is one of the best tools for the investigation of oriented monolayers behaviour. Recently, lipid monolayers have been extensively used as membrane models because of their homogeneity, planar geometry, and specific orientation, and also because Langmuir films allow rigorous thermodynamic analysis [66,67,81]. In order to prepare a model membrane, it is necessary to dissolve lipid amphiphiles in a water-insoluble solvent and deposit it on a water surface (subphase) with a microsyringe. The subphase can be either pure water or an aqueous buffer solution (unlimited ability to control the pH of the environment). As the solvent evaporates, the lipid amphiphiles are oriented to minimise contact of their nonpolar regions with water while maximising the water contact of their polar regions. In this way, a one-molecule-thick lipid film is formed as a monolayer, which provides a useful model system for studying the lateral packing interactions of lipids in each leaflet of a biomembrane [82]. A Langmuir monolayer represents half of a biological membrane (Figure 2); therefore, it is less suited to study transmembrane processes, although it can certainly be applied to mimic processes taking place at membrane surfaces.

Moreover, in order to achieve a better simulation of the biological membrane, it is possible to form multicomponent lipid monolayers containing proteins, sterols, or lipopolysaccharides (LPS). During the determination of the quantitative affinity of antibiotics to the membrane surface, it is important to form membranes containing LPS because the outer membrane of G- bacteria is made primarily of this substance. LPS molecules contain the hydrophobic lipid A region, the core oligosaccharide, and the O-antigen polysaccharide. Lipid A consists of the backbone (phosphorylated *N*-acetyl glucosamine dimer) with up to seven attached fatty acids, which form the apolar inner core of the outer membrane and serve as hydrophobic anchors for the branched sugar chains (Figure 2). The term LPS defines the full molecule but commonly corresponds only to a portion of the head group of molecules in Langmuir monolayer studies.

**Figure 3:**
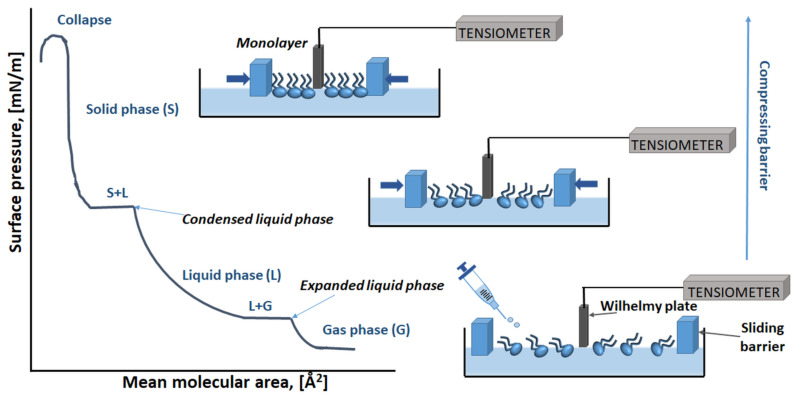
Surface pressure–area isotherms (π–A) of a Langmuir monolayer and molecules in different phases.

The Langmuir monolayers offer a unique advantage which is the possibility to achieve varying density and composition of lipids at the interface in a controlled manner so that the energetics can be studied via surface tension measurements [83]. The surface tension of water is caused by different entropy of water molecules at the surface and in the subphase. Water molecules tend to maximise hydrogen bonding with neighbouring water molecules and also to limit their contact with the air molecules. As a result, water molecules are pulled toward the bulk subphase, which generates surface tension. The addition of an amphiphile to the water surface causes changes in the surface tension, which provides information about lipid–lipid and lipid–water interactions. The Langmuir monolayer technique enables the formation of a lipid film on the water subphase and characterisation of lipid–lipid, lipid–water, or lipid–drug interactions on the basis of compression isotherms, obtained by measuring the surface pressure (π) of the interfacial monolayer as a function of the mean molecular area (A). Two automated barriers moving along the subphase surface ensure the appropriate rate of film compression. Upon compression of the monolayer, the surface pressure (π) is measured continuously as a function of average molecular area (A) at a constant temperature guaranteed by the thermostating of the Langmuir trough. The surface area is reduced upon the compression (Figure 3) and forces the appropriate reorganisation of molecules at the interface, which means that the obtained two-dimensional insoluble monolayer undergoes different physical states.

These physical states are related to the level of conformational order of the molecules at the interface, which is implied by the presence of intermolecular interactions within the monolayer. With increasing surface pressure, the density of the monolayer packing increases until the collapse point is reached (π_collapse_), above which it is not possible to increase the pressure any further. Analysis of the π_collapse_ values can be used to determine the stability and the miscibility behaviour between the components in a mixed monolayer [84]. The course of the π–A isotherm allows researchers to obtain information about the interactions of molecules at the interface. The observed phase behaviour of the monolayer is determined mainly by the physicochemical properties of the lipid amphiphile, the subphase temperature, and the subphase composition. The two most commonly observed monolayer states, the liquid-expanded (LE) and liquid-condensed (LC) ones, are analogous to the liquid–crystalline and gel states in bilayers, respectively [85]. Moreover, a lipid monolayer is also characterised by changes in terms of two-dimensional compressibility (*C_s_*) and it is usually expressed as the surface compressional modulus (*C_s_*^−^^1^). *C_s_*^−^^1^ was originally defined by Davies and Rideal [86]; this parameter is more responsive to subtle changes in the monolayer structure during lateral interactions; therefore, on the basis of its values, it is possible to conclude about the lateral packing elasticity within the monolayer system,

(1) $$C_{s}^{-1}=-A\left(\frac{d\pi }{dA}\right)$$

In general, the higher the maximum compressibility modulus value is, the greater the increase in the rigidity (i.e., a decrease in the compressibility) of the monolayer will be.

The asymmetric orientation of lipid and water molecules at the air–water interface generates a sizeable (hundreds of millivolts) electrical potential perpendicular to the plane of the interface. For this reason, currently, the Langmuir trough is equipped with a surface potential sensor. The interfacial potential is measured and recorded simultaneously with the surface pressure as the movable barrier compresses the lipid monolayer. The obtained curve showing the changes in potential vs. area per molecule (or surface pressure) complements the data inferred from the π–A isotherm and provides information about the monolayer composition, molecular orientation, degree of molecular dissociation, and molecular interactions at the interface. Knowledge regarding the surface potential is particularly important when studying the mechanism of interactions between the dipolar structure of LPS monolayers and the antibiotics with charge or strong dipoles. The experiments including surface potential measurements are expected to disclose the reason why the presence of some antibiotics results in the disappearance of the shoulders on the π–A isotherms of monolayers containing LPS.

**Figure 4:**
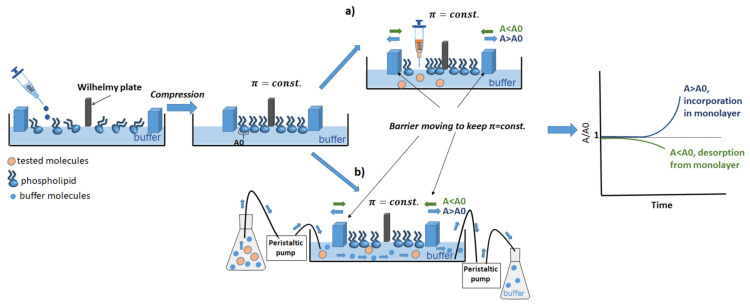
Relaxation of a monolayer by Langmuir technique: (**a**) injection underneath a monolayer into the bulk of the subphase; (**b**) subphase replacement.

Moreover, the use of Langmuir trough in combination with a Brewster angle microscope (or with a fluorescence microscope, AFM) allows for the precise imaging of the monolayer morphology upon the film compression, visualisation of the interfacial organisation of lipid constituents of the monolayer, or the changes in the interfacial behaviour resulting from incorporation of the tested compound into the monolayer. To gain a better insight into the antibiotic–membrane interactions, ATR–FTIR spectroscopy monitoring can be applied [87]. In order to obtain more information about the incorporation of a given substance into a membrane, it is possible to study the relaxation of a monolayer. For this purpose, a monolayer is spread at the air–water interface and kept at a predetermined constant surface pressure (Figure 4a). This method is called the constant surface–pressure approach. A known amount of a concentrated solution of a given substance (which dissolves in it) is injected underneath the monolayer. and the insertion of this substance is monitored by measuring the film expansion; the increase in the area of the film is linearly proportional to the number of inserted molecules.

The simplification of the membrane system is essential for the investigation of specific interactions at the molecular level in the membrane structure. On the other hand, it can also interfere with the accurate understanding of some membrane functions.

When water-insoluble biomolecules are investigated, the approach is based on making a mixed Langmuir monolayer by co-spreading of membrane components and biomolecules onto the water surface [88]. By changing the proportions of monolayer components and analysing the stability and miscibility of the investigated mixed system, it is possible to infer the nature and strength of interactions between the film molecules [89]. A newly proposed method involves the replacement of the subphase with a buffer containing various tested substances using a dosing pump. This approach allows for delivering new substances to the system and changing the experimental conditions during the process, e.g., the pH of the subphase. The subphase flow must be slow enough not to disturb the continuity of the monolayer. The proposed method may be useful in studying the interactions of monolayers with proteins and metal ions, which affect the stability of Langmuir monolayers (Figure 4b). In our earlier report, we have investigated the interactions of saponins, nitrofurantoin (NFT), and their mixtures with the model phospholipids monolayer by replacing the water subphase with a buffer with dissolved tested substances [90]. In that study, we also measured the relaxation of a model membrane by the formation of a phospholipid monolayer on a buffer subphase and then we exchanged the subphase to deliver saponins or/and NTF to the investigated system, using a peristaltic pump (Figure 4b). The subphase flow was slow enough to neither disturb the structure of the monolayer nor cause its destruction in the process of replacement of the bulk phase with a new one. The relaxation experiment consisted of keeping the surface pressure (π) constant and recording the area (A) as a function of time. If the value of (A(t)) is greater than A0 (A(t)/A0 > 1), an increase in the area per molecule occurs, which is caused by the embedding of the tested molecules into the phospholipid monolayer. Otherwise, if A(t)/A0 < 1, there is a surface area loss in the monolayer, and therefore, it can be assumed that the phospholipid molecules desorb from the monolayer and dissolve in the subphase. The A/A0 parameter was, therefore, a measure of the monolayer stability. On the basis of the data in our study, nitrofurantoin was found to slightly interact with the phospholipid POPE monolayer, leading to a reduction of the area per molecule over time, which was consistent with literature reports [91]. Moreover, we also assumed that domains of saponins could be formed in the POPE monolayer, and these structures may become embedded in the monolayer, consequently changing the fluidity of the membrane. In [90], we proposed the most probable mechanism of saponins and NFT interactions with the bacterial cell membrane, inferred mainly from the relaxation experiments of a model membrane. Moreover, in the studies on the monolayer membrane models, it is also possible to simulate the actual biological conditions, as well as temperature and pH. The monolayer systems are used for protein or peptide interaction studies [92,93] and also drug penetration studies [81,94].

## 5. Langmuir Monolayer as a Model Bacterial Membrane

The Langmuir technique is also gaining increasing recognition in basic research on biodegradation processes, undertaken in order to understand the mechanism of microorganisms’ action. The Langmuir monolayer technique enabled the construction of models of soil bacteria membranes differing in the mutual ratio of the main phospholipids [95,96]. The model membranes are simulated by single phospholipids such as DMPE, DMPG, POPE, DPPE, DPPG, DOPG, as well as their mixtures at various molar ratios [96,97]. Many experiments have been performed on model bacterial membranes, most often simulated by monolayers composed of mixtures of phospholipids such as DMPE, DMPG, and CL at different proportions [98]. The interactions of antibiotics with model bacterial membranes with the use of *E. coli* extract, LPSs, or dioleoylphosphatidylcholine (DOPC) dissolved in CHCl_3_ solution have been reported in [64,99,100]. The contents of individual phospholipids in the membrane are very important because changes in the composition of acyl chains or head groups impact the bilayer fluidity and stability; consequently, they affect the membrane response to environmental perturbations [101]. According to some literature reports, the greater contribution of longer acyl chains of fatty acids leads to a decrease in membrane fluidity [102]. The lipids with longer fatty acid chains are engaged in more interactions with the hydrophobic fatty acid tails, which stabilises the crystal-like state and makes the membrane less fluid. However, the precise roles played by membrane phospholipids in bacterial physiology and stress adaptation have not been not fully elucidated. The Langmuir technique can bring us closer to understanding the mechanisms of these processes because the manipulation of the phospholipid composition in the monolayer is easy and allows for the determination of specific roles of phospholipids on the molecular level [83,90,103]. Rowlett et al. [104] have examined *E. coli* cells in which the phospholipid composition of the membrane can be systematically manipulated. They have found that alterations in the content of PE or CL lead to modifications in the cellular envelope structure and composition, membrane biogenesis, and homeostasis pathways. Additionally, phospholipid-altered strains exhibit perturbations in surface adhesion and change the susceptibility to environmental stresses, which indicates that the maintenance of proper membrane phospholipid composition is critical for sufficient bacterial adaptation. Their results suggest that modification of the phospholipid composition changes the size and complexity of the long-chain oligopolysaccharides. In particular, the absence of PE dramatically reduces the number of O-antigen repeats. Moreover, it was shown that PE can only be partially replaced by PG [104], leading to the formation of less complex LPS. The same authors have demonstrated that an incremental increase in PE alters the LPS structure. LPS analysis has shown that with an increase in the amount of PE, the number of O-antigen repeats increases. In contrast, the lack of cardiolipin CL caused a different O-antigen repeat pattern, with longer LPS chains [104].

## 6. Modelling of Cellular Membrane for Antibiotic Biodegradation Studies

Bioaugmentation is one of the biodegradation methods of organic pollutants in the environment. This technique involves the inoculation of selected species of microorganisms into the polluted soil. Unfortunately, bioaugmentation is frequently ineffective because the introduced microorganisms die out due to their low resistance to exogenous phospholipases (PLC). Phospholipases cause hydrolysis of the ester bond present in the structure of the phospholipids, making scaffolds of the bacterial membrane and destroying the cellular membranes of the microorganisms. Therefore, when carrying out bioaugmentation processes, it is essential to understand the interaction of PLC with different classes of phospholipids. The interactions of bacterial PLCs with phosphatidylcholines and sphingomyelin have been studied with the use of different membrane models, including Langmuir monolayers [105,106,107]. Experiments were conducted in two variants. In the first, the monolayer was compressed to the required surface pressure, and next, a solution of PLC was injected into the subphase. Then, the temporal surface pressure changes were monitored in the presence of PLC while maintaining a constant area between the barriers. In the second approach, the surface pressure was stabilised at a constant level, and the evolution of the mean molecular area A in time was followed. On the basis of the study on the interaction of phospholipases with different types of phospholipids, a correlation was found between the hydrocarbon composition and the phospholipid class and PLC activity. Knowledge of these interactions is vital for taking proper decisions regarding the selection of microorganisms for a given process of bioaugmentation, based on the determination of the bacterial membrane compositions. When the bacterial membranes show the highest content of the phospholipids resistant to this lipase, the bacteria are expected to be effective in the process. Broniatowski et al. [96] have proposed the following order of phospholipids: PC » PE > PG » CL according to the strength of interactions with PLC. The above ordering of phospholipids is generally consistent with the data published previously for other model systems [107]. According to this order, Gram-positive bacteria would be the most resistant to the secretory PLCs and lipases present in the soil, as their membranes are rich in cardiolipins and/or phosphatidylglycerols, compounds less susceptible to the action of phospholipases.

Another interesting example of the use of the Langmuir method is the investigation of the interactions of LPS with antibiotics. As is known, Gram-negative bacteria possess the outer membrane (OM), which makes a high resistance barrier for the permeation of many antibiotics, even those that have been proven effective against Gram-positive bacteria [108]. Nevertheless, certain classes of antibiotics are more effective against Gram-negative bacteria, implying that their permeation might involve specific interactions with the components of the outer membrane to facilitate their uptake [109,110]. Lipopolysaccharides (LPS), present in the outer leaflet of the Gram-negative membrane, are considered to be the major impediment for antibacterial agent permeation. As shown [111], the presence of divalent cations significantly affects the transport of compounds through the LPS monolayer because divalent cations allow for the formation of lateral bridging to neighbouring LPSs and finally develop a cross-linked structure. This leads to the formation of a more condensed layer, provides additional rigidity, and minimises the occurrence of defects that may favour the permeation of a variety of molecules. Finally, a strong barrier to both hydrophilic and hydrophobic compounds, including antibiotics and peptides, is built. Moreover, it has been shown that bacterial membranes containing LPS devoid of the O-antigen exhibit higher sensitivity to detergents and antibiotics, compared to those with longer polysaccharide chains [112]. It has been reported that the OM of Gram-negative bacteria acts as a relatively effective permeability barrier against most hydrophobic antibiotics [97]. For hydrophilic antibiotics, a variety of passage mechanisms through the outer membrane have been presented in the literature. It has been shown that small molecules permeate through porins (as β-lactams), while those larger than the 1.2 nm effective cut-off diameter are generally excluded [113]. On the other hand, for some groups of antibiotics (as polymyxins), a mechanism including displacement of divalent ions, and thus, disruption of the membrane integrity has been indicated. It has been shown that positively charged polymyxin accumulates at the bacterial outer membrane due to electrostatic attraction to LPS, where it displaces the divalent ions that condense LPS molecules into their tightly packed lamellar structure. Information on the strong binding of LPS to the polymyxin B antibiotic can be found in [114,115]. However, the actual affinities of major antibiotic classes towards LPS have not yet been determined. Understanding these interactions can provide insight into the biodegradation mechanism and help develop and improve the bacterial consortia that would be effective in the removal of contaminants such as pharmaceuticals. The Langmuir technique also allows for quantification of the interactions between LPS and antibiotic molecules at different degrees of monolayer compaction. If the antibiotic molecule interacts with the lipid layer, it is inserted in it and leads to the monolayer expansion. The controlled density of monolayer packing provides the antibiotic molecules access to all parts of lipid molecules through a range of compression states and also allows estimation of the molecular insertion area. Cethuk et al. [116] have applied the Langmuir monolayer method to confirm that the structure of the antibiotic has a significant impact on the incorporation mechanism of the compound in the model membrane. They have revealed that the hydrophobicity of the antibiotic is a desirable feature which helps in its insertion and retention inside the aliphatic slab of LPS over a range of pressures. The incorporation of polycationic gentamicin, polymyxin B, or colistin into the monolayer causes expansion of the monolayer area which does not decrease with pressure, suggesting that they stably separate the LPS molecules and possibly create voids in the hydrophobic slab. These defects increase the permeability of the membrane. Moreover, on the basis of the Langmuir technique, it has been shown that these antibiotics do not undergo hydrophobic insertion but are instead strongly driven into the polar LPS layer by electrostatic interactions in a pressure-independent manner. The character of the π–A isotherms obtained for the system of polymyxin–LPS monolayer indicates that this antibiotic does not incorporate into the LPS monolayer in the relaxed state, but it interacts with the polar groups of LPS in a way preventing the increase in their density upon the monolayer compressing. The measurements using the Langmuir technique have also confirmed that the polar/electrostatic mode is obviously more disruptive for the LPS layer, which explains why aminoglycosides and polymyxins are more effective and specific against Gram-negative bacteria. Moreover, based on the data obtained with the Langmuir technique, the authors of [116] have proposed some simple criteria to predict which compounds will cross the membrane. It has been suggested that if an antibiotic can hold a detectable mole fraction among LPS molecules during compression all the way to reach the collapse pressure, then it can be expected that this drug would likely permeate through the native bacterial membrane.

The use of the Langmuir monolayer method provides data which enable a detailed description of the interactions of bacterial membrane components with antibiotics [64,117] such as pentacyclic triterpenes [118], nitrofurantoin [90,91], vancomycin [119], and antimicrobial peptides [93,120,121]. With the use of the Langmuir technique, it is possible to obtain the data that will shed light on the complexity of bacterial membrane–antibiotic interactions and be very helpful in tackling the antibiotic resistance [122], which is also an important problem in the biodegradation process [123].

## 7. Conclusions and Future Perspectives

The Langmuir monolayer technique is one of the most precise and simple methods to form high-quality ordered monolayers which imitate a leaflet of a bacterial membrane and control its composition. This technique has been successfully applied to study the properties of biomembranes, various processes occurring at the membrane, as well as molecular-level interactions between membrane components such phospholipids, proteins, or antibiotics. As expected, the interactions between an antibiotic and a lipid surface bring significant biological consequences which may include effects on the morphology and dynamics of the lipid monolayer and thus on the properties of the bacterial membrane. The Langmuir monolayer relaxation study allows for the identification of changes in the lipid–antibiotic film morphology and the organisation of the monolayer components at the interface of the lipid monolayer and subphase. Moreover, the method of monolayer relaxation permits estimation of the affinity of an active substance to lipids by direct monitoring of the monolayer area expansion, within the constant surface–pressure approach. In our opinion, this technique is a promising tool for prognostic evaluation of the interaction of antibiotics with components of real membranes. The information provided from examining the impact of an antibiotic on the monolayers that consist of individual components of the cell membrane is expected to help predict the interaction of this antibiotic with the actual cell membrane. Moreover, the data obtained by using the Langmuir technique permit a deeper understanding of the interaction mechanisms of antibiotics with the bacterial membrane at the molecular level. In combination with spectroscopic techniques, the Langmuir method may help to establish preferred antibiotic orientations and the depths of its insertion into the cell membrane.

This mini-review should be treated as a reference document for scientists who want to be introduced to the structure of bacterial membranes and learn more about the Langmuir technique. The readers are invited to find more details on the topics presented in this mini-review in the provided references.

## Figures and Tables

**Table 1:** Main phospholipids constituents of bacterial membranes (according to [65]; ‘+’ indicates that given phospholipid was identified in several representants of mentioned bacteria phylum).

	Alpha-Proteobacteria	Beta-Proteobacteria	Gamma-Proteobacteria	Delta-Proteobacteria	Epsilon-Proteobacteria	Cyanobacteria	Actinobacteria	Spirochetes	Planctomyces	Firmicutes
PG-phosphatidylglycerol	+	+	+	+	+	+	+	+	+	+
CL-cardiolipin	+	+	+	+	+	+	+	+	+	+
PS-phosphatidylserine				+	+					
PE-phosphatidylethanolamine	+	+	+	+			+	+		+
MMPE-monomethyl PE	+		+							
DMPE-dimethyl PE	+		+							
PT-phosphatidylthreonine				+						
PC-phosphatidylcholine	+		+						+	
PA-phosphatidic acid									+	
GPL-glycophospholipid										+
LPG-lysyl-phosphatidylglycerol	+	+	+	+						+
APG-alanyl-phosphatidylglycerol										+
LCL-lysyl-cardiolipin										+

**Table 2:** Characteristic of biomimetic model membranes.

Model of Mimic Biological Membrane	Characteristic of Model
Langmuir monolayer (Monomolecular insoluble lipid films) 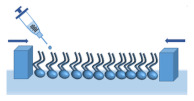	- Quite easily prepared technique from each type of membrane lipids; monolayer can be considered as a half the bilayer of biological membranes (as two-dimensional systems). - Easy contol of the packing of the spread molecules, the composition of subphase (pH, ionic strenght) and temperature. - Excellent model for investigating the insertion of amphipathic compounds into the membrane. The mian disadvantage: lipid monolayers are constituted of only one lipid leaflet and therefore do not reflect the complexity of biological membrane structure.
SLB (A flat lipid bilayer supported onto a solid surface) 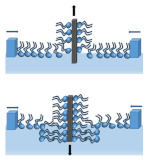	- Model membrane prepared quite easily; SLB are much more stable than lipid vesicles. - The composition and the lipid asymmetry of SLBs can be controlled. - Excellent method to predict the molecular organization of biological membranes and the phase behaviour of membrane, for studying the molecular interactions of drugs with cell membranes and interactions of membrane-protein systems. The main disadvantage: the solid substrate may affect the membrane properties of the biomimetic system such as incorporation of substance into membrane or the mobility of membranes components.
Liposomes (Lipid vesicles) 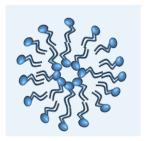	- The preparation technique of lipid vesicles is more complex and selective. - Liposomes composed of two lipid leaflets which are better mimic iological membranes than Langmuir monolayer. - Excellent method for studying membrane phase behavior and membrane processes: membrane fusion, molecular recognition, cell adhesion, membrane trafficking. The main disadvantages: Before using the liposome as a membrane model, its the final composition should be determined as it may differ from the composition of the initial lipid mixture used to form vesicles. It’s difficult to control lipid asymmetry when using vesicular model systems.

## Data Availability

Not applicable.
